# A Standardised Protocol for Evaluation of Anthelminthic Efficacy

**DOI:** 10.1371/journal.pntd.0001010

**Published:** 2011-03-29

**Authors:** John Horton

**Affiliations:** The Paddock, Hitchin, United Kingdom; London School of Hygiene & Tropical Medicine, United Kingdom

Although the concept of targeted or community-wide treatment for soil-transmitted helminths (STHs) is not new, having been initiated originally by the Rockefeller Sanitary Commission in the United States [1], [2] and more recently supported by Warren [3] and others some 30 years ago as a feasible approach, it is only in the last 10 years, since the World Health Assembly resolution [4], that helminth control has become a reality. Today there are a number of global initiatives, such as the Global Programme for the Elimination of Filariasis and the Schistosomiasis Control Initiative, where anthelminthics form a large part of the structure of mass treatment programmes. In addition, the move towards integrated programmes and a number of different drug interventions has meant that, at last, a large portion of the global population at risk is being treated. This was further augmented in 2010 by additional large donations from GlaxoSmithKline [5] and Johnson & Johnson [6], so that in the near future almost one quarter of the global population may be treated each year with anthelminthics. However, for the STHs, as for schistosomiasis, these interventions are based on a very limited drug armamentarium. There is therefore a very real risk that widespread use may engender drug resistance and put the programmes at risk. Thus, it is imperative that active monitoring is undertaken to detect, and hopefully respond to, the first signs of drug resistance. While the genetic changes associated with drug resistance to the most widely used drugs for STHs, the benzimidazole carbamates, albendazole and mebendazole, are well understood, active screening using genetic markers is probably impractical other than for confirmation of its occurrence. Therefore, monitoring of drug efficacy in the field is, and will probably remain, the tool of choice for the foreseeable future.

For field monitoring of anthelminthic efficacy, it is essential that standard protocols are employed that have been tested for their sensitivity and ease of implementation. Since the benzimidazoles were introduced for human use some 30 years ago, there have been many studies, mostly looking at efficacy in single sites on a single occasion, although the work in Pemba Island, Republic of Tanzania, has looked at longitudinal changes in efficacy as well [7]. The problem underlying most of these studies is the lack of a consistent methodology, a point noted in a Cochrane analysis of the nutritional and cognitive impacts of anthelminthics [8] and more recently by Geary et al. [9]. Thus, the paper by Vercruysse and colleagues in this issue [10] is a welcome attempt at developing a rational and tested methodology for assessment of albendazole (and therefore, by inference, anthelminthic) efficacy.

They have opted to use the McMaster technique, which, while being better for quantitation, is more difficult to use in the field compared to the more widely used Kato-Katz test. The latter is, however, only semi-quantitative and needs to be read immediately to identify hookworm eggs. A change to the McMaster or an equivalent quantitative technique is necessary if one is hoping to track alterations in anthelminthic efficacy over time that will require the more sensitive evidence from egg reduction measures rather than gross cure rates While cure rates are usually considered to be a key measure of efficacy, egg reduction is of greater importance in STH control, because the aim is to reduce infection, rather than to eliminate it, since high worm burdens are the cause of morbidity. Therefore, drug failure, as shown in lower egg reduction rates, will have greater importance in control approaches, and will occur earlier than poor cure rates. Currently, however, cure rates will remain the key measure of anthelminthic efficacy for new drugs, and appropriate thresholds need to be agreed upon in line with those (cure rates >90%) used for approval of veterinary medicines. The authors argue that new efficacy levels need to be agreed to replace those currently accepted by the World Health Organization [11], but this may need further study before agreement can be reached.

In establishing the rationale for their study, the authors argue strongly for numbers that will be statistically sound. Regrettably, one of the weaknesses in the data is that three countries failed to reach their overall recruitment goal, and since three countries had more than one site under study, it is possible that only two countries exceeded the recruitment goal in terms of site recruitment. Interestingly, the two worst performing countries were Brazil and India, where one would expect to be able to find suitable sites to test efficacy. It also emphasises the problems in conducting efficacy studies, and recruitment is likely to be an increasing problem as control programmes become more widespread. Additionally, since infection rates vary widely, both globally and locally, it is essential that longitudinal measures of efficacy be obtained from defined sentinel sites. The level of detail provided in the paper (district/province/state) is clearly inadequate for follow-up, especially when more accurate identifiers such as global positioning system coordinates are available.

A standard evaluation protocol is a fine goal, but it is unlikely that the current approach will gain universal acceptance immediately. The methods will need to be fine tuned, and this requires at least a repeat of the current study, ensuring that recruitment numbers are consistent at all sites. For evaluation of efficacy for a particular helminth, defined thresholds of recruitment should be reached; from the paper by Vercruysse et al. it appears that 200 individuals per site and species would be sufficient. Basing such critical evaluations on small numbers is a concern, especially as findings of reduced efficacy could result in costly changes in policy or approach.

Finding appropriate locations to use for long-term evaluation is now a problem, and will become more difficult with time. It is therefore imperative that the lessons from this study are taken forward and new studies started to consolidate the approach and provide the material to argue the case. The main difficulty now will be convincing the world that there is a single acceptable approach to testing anthelminthic efficacy, both for public health monitoring and for drug registration. Unfortunately, scientists are often very conservative when it comes to changing methods, and it will require effort on the part of the World Health Organization and other public health bodies to drive through a standardised anthelminthic protocol as being essential to long-term public health goals. To fail to do so will mean that resistance may only be identified when it is too late, and that will be failing those who need anthelminthics most.
